# A bioassay method validation framework for laboratory and semi-field tests used to evaluate vector control tools

**DOI:** 10.1186/s12936-023-04717-w

**Published:** 2023-09-28

**Authors:** Agnes Matope, Rosemary S. Lees, Angus Spiers, Geraldine M. Foster

**Affiliations:** 1 Vector Biology Department, Liverpool School of Tropical Medicine, Pembroke Place, Liverpool, L3 5QA UK; 2 Innovation to Impact (I2I), Liverpool School of Tropical Medicine, Pembroke Place, Liverpool, L3 5QA UK

**Keywords:** Vector, Bioassay, Insecticide, Evaluation, Validation, Verification, Precision, Variability, Feasibility, Insects, ITN, Control

## Abstract

Vector control interventions play a fundamental role in the control and elimination of vector-borne diseases. The evaluation of vector control products relies on bioassays, laboratory and semi-field tests using live insects to assess the product’s effectiveness. Bioassay method development requires a rigorous validation process to ensure that relevant methods are used to capture appropriate entomological endpoints which accurately and precisely describe likely efficacy against disease vectors as well as product characteristics within the manufacturing tolerance ranges for insecticide content specified by the World Health Organization. Currently, there are no standardized guidelines for bioassay method validation in vector control. This report presents a framework for bioassay validation that draws on accepted validation processes from the chemical and healthcare fields and which can be applied for evaluating bioassays and semi-field tests in vector control. The validation process has been categorized into four stages: preliminary development; feasibility experiments; internal validation, and external validation. A properly validated method combined with an appropriate experimental design and data analyses that account for both the variability of the method and the product is needed to generate reliable estimates of product efficacy to ensure that at-risk communities have timely access to safe and reliable vector control products.

**Supplementary Information:**

The online version contains supplementary material available at 10.1186/s12936-023-04717-w.

## Background

Vector control interventions play a fundamental role in the control and elimination of vector-borne diseases, due to their effectiveness in preventing infection and reducing disease transmission [1–3]. Products for use in vector control interventions undergo a standardized process through World Health Organization (WHO) pre-qualification to ensure each product meets specified standards [4–7]. For many years vector control relied upon insecticide classes (pyrethroids, organochlorines and carbamates) that induce rapid neural intoxication and death [8]. Therefore, during the laboratory phase of this evaluation, product characteristics are assessed using bioassays that typically measure the knockdown and mortality of exposed insects, usually mosquitoes. High heterogeneity in measured outcomes and poor agreement between methods have been observed [9–14]. The observed variability in bioassays is exacerbated by the high tolerance ranges for the insecticide content of insecticide-treated nets (ITNs), because ITNs are non-homogeneous products that can vary within and between each panel used for net construction [7, 15]. Similarly, the variability of spray quality during indoor residual spraying (IRS), and the 50% tolerance range for the concentration delivered to wall surfaces, increases the observed variation in bioassay results over and above the variability of the bioassay endpoints themselves [16].

New classes of insecticide are now available for use on ITNs and for use in indoor residual spraying (IRS). Therefore, new and sufficiently sensitive bioassay methods are required to assess the characteristics of products treated with novel active ingredients (AI) against vector populations [17]*.* These new bioassay methods may use endpoints other than knockdown and 24 h mortality, which were endpoints chosen to demonstrate the rapid toxic action of the pyrethroid insecticide class. A method refers here to a new or modified bioassay, a technique using commercially available or in-house reagents, a prototype kit or a semi-field test. Bioassay method development requires a rigorous validation process to ensure that the methods are able to use entomological endpoints to accurately and precisely describe product characteristics within the manufacturing tolerance range currently specified by the WHO for that product [15]. This requires validation of the method’s quality, reliability, and consistency of results prior to implementation in multiple facilities [18].

Method validation is a process that is used to demonstrate that a technique is suitable for its intended purpose and that the results obtained are reliable [19, 20] Experimental design for method validation is centred on defining and measuring analytical error [21, 22]. These analytical errors are broadly categorized into random errors or imprecision, which refers to the distribution of test values, and systematic errors or inaccuracy, which is a shift or bias between the central measure value and the correct value [22]. The total error is a combination of random and systematic errors that plays a vital role in assessing the test’s performance based on the allowable measurement error defined during the preliminary development and feasibility stages.

Although standardized method validation guidelines for assessing novel methods exist within the healthcare and pharmaceutical industries (among others) [23–25], standardized validation practices are not typically used in the vector control field. This is partly because the widely used validation study design methodologies in use in other fields are not necessarily directly transferable to bioassays using live biological material or to tests performed in variable field conditions, resulting in a lack of relevant method validation guidelines [25, 26].

Many vector control product testing laboratories and trial sites are Good Laboratory Practice (GLP)-accredited facilities, which is a managerial quality control system for laboratories that regulates the planning, performance and reporting of studies conducted in non-clinical laboratories [27, 28]. The implementation of standardized method validation processes is independent of GLP accreditation, and therefore these standardized processes can be implemented in all laboratories that design and develop bioassays.

This report describes a framework for bioassay method validation and presents case studies for its use in bioassay development for vector control products. The framework draws on formalized validation processes used in other scientific disciplines and adapts them into a modular approach that recognizes the unique nature of bioassays that use entomological endpoints to characterize the insecticidal properties of vector control tools. The framework is suitable for use to validate methods that might be used in product testing, non-bioassay methods that might be used for routine quality surveillance, and for other bioassays with an entomological endpoint, such as resistance monitoring or methods used to test non-product-based vector control. It may also be used to think through issues that are causing variability in bioassays within a product testing facility. Within each process stage, validating laboratories can select the most relevant sub-studies to conduct based on the intended purpose of the method under development or in use and apply the framework to evaluate method precision, accuracy and replicability of both laboratory and semi-field tests.

## Method validation framework design

Method validation and method verification are two distinct processes that may be required at different points in the development of a method from initial design to implementation in multiple laboratories and/or semi-field sites. A laboratory is required to carry out validation when:

i. A laboratory has designed or developed a new method
ii. A laboratory is required to demonstrate comparability between a novel method and an existing standard method.
iii. A standard method has been modified
iv. A standard method is used for a new purpose

Full laboratory design and development involve the conception of the method from scratch, including preliminary testing to define whether the method is logistically feasible and can measure the desired outputs. Where the novel method measures the same outputs as an existing method, the novel and existing methods should be compared during validation [22, 25]. Modification of an existing standard method alters one aspect, for example, extending the exposure time used in a test and requires the internal and external validation process stages (see below) to be repeated. To use a method for a new purpose might involve the use of a method validated for use with one product class being used to characterize a different product class. Validation in such cases is dependent on the magnitude of the change but may require conducting feasibility experiments to demonstrate that the change in scope has not affected the capacity of the method to reliably capture its endpoints [26, 29].

Laboratories adopting a validated method should conduct method verification [22], which can be conducted using controls of a known value and/or response and ensures that the implementing laboratory can reproduce the established method performance.

### Stages of the method validation process

Four stages for evaluating bioassays and semi-field tests are proposed: (1) preliminary development; (2) feasibility experiments; (3) internal validation, and (4) external validation (Table 1). The stages are designed to ensure that the method is scientifically sound and reproducible within the variation exhibited in biological tests [5, 22]. During preliminary development, the method is devised, and endpoints and analytical requirements are defined and tested [25]. At the feasibility stage, the performance parameters and endpoints are verified, and a standard operating procedure (SOP) is drafted. In internal validation, the analytical performance of the method is tested, the method claim is drafted and a data package for external validation sites is compiled. During external validation, the method is evaluated in multiple laboratories/sites and the final method claim is produced. Once external validation is successful, the method can be implemented.

**Table 1:** Validation process stages for methods used to assess vector control tools

	(1) Preliminary development	(2) Feasibility experiments	(3) Internal validation	(4) External validation
Method Goal	Method designed/ modified and method performance solidly understood	Scientifically sound, repeatable and efficient method	Minimally validated method	Fully validated method
Analytic Goals	• Build robustness into the method • Ensure repeatable and reliable data generation	Establish results for appropriate validation elements	• Limited method comparison in a single site • Defined method performance characteristics at a single site	• Extensive evaluation of method in multiple sites • Defined method performance characteristics at several sites
Documentation Requirements/Output	Initial method validation study protocol • Define the design, application, purpose, and scope of the method • Define the endpoint(s) • Define the analytical requirements (performance parameters and acceptance criteria) • Define testing conditions • Define controls • Select comparator method (where applicable) • Conduct baseline experiments	Draft SOP for the method • Define the final endpoint (s) for validation • Define strains for validation • Define testing conditions for conduct of validation • Propose a suitable experimental design for validation (sample size, testing pattern, and testing period as necessary) • Verify relevant performance characteristics (compare performance parameters results to the pre-specified acceptance criteria)	Internal validation protocol and SOP for the method • Analytical requirements stated • Method claim drafted • Identify laboratories/sites for external validation • Compile a data package	External validation protocol and report with the method ‘fitness for purpose’ statement • Method final claim produced • Analytical requirements stated • Implementation can be recommended

^*^Adapted from [24, 34]

### Preliminary development

The purpose of the preliminary development stage is to assess the proposed method design for suitability for a defined purpose in a defined setting, define the endpoints and the level of allowable analytical error (both imprecision and inaccuracy) for each, and to build robustness (minimize the impact of changes in variables or testing conditions on results) into the method. Experiments conducted as part of preliminary development typically use small sample sizes so that data on a range of conditions and variables (for examples, refer to the testing conditions below) can be generated and used to refine the method parameters and guide the experimental design for feasibility and internal validation.

#### Define method scope and endpoints

The method design, application, and endpoints to be used to assess method performance should be clearly defined. An endpoint is a quantifiable output that can be recorded using the method, e.g., oviposition inhibition in female mosquitoes exposed to an insect growth regulator. Every endpoint that is intended to become part of the eventual method claim must be clearly defined. The definition should state precisely what is to be measured, when, and the desired range of measurement, *e.g.,* number of eggs laid per female, up to five days post-exposure, from 0 to 300 eggs’.

#### Define acceptability criteria

Acceptability criteria define the allowable error within the method and are dependent on the effect size of each endpoint. In the example from the previous section, for the measurement outcome: *‘50% reduction in the number of eggs laid per female, up to five days post-exposure, from 0 to 300 eggs’ an acceptability* criterion might be: *‘Measure a 50% reduction in the number of eggs laid with 10% precision within the reportable range’.* The allowable error should be as small as possible yet align with what is practically achievable and scientifically justifiable [30]. In some cases, published or internal data from similar methods can be used to estimate the desired magnitude; for entirely novel methods, this may not be possible and can be refined following feasibility experiments.

##### Factors to consider when defining acceptability criteria:

- Within-day imprecision should be less than ¼ of the total allowable error or the coefficient of variation (CV) < 20% [22, 31, 32]. Between-day imprecision typically has the same error level, but can be increased if justified [25]
- For measurement outcomes relating to target values, criteria can be set either as a multiple of standard deviation (SD), *e.g.*, within 3SD of the mean, or within a percentage range of the target value *e.g.,* ± 25%
- For phenotypic measurement outcomes, an indicative threshold can be used although such thresholds should be used with care [33]. For example, 98% mortality in a susceptibility test using a discriminating concentration in monitoring for insecticide resistance.

#### Identify the analytical parameter/s to be measured

At least one analytical parameter must be evaluated [20, 23]. Within the common analytical parameters of accuracy/trueness, precision, linearity, range and robustness, the most useful parameters for bioassay and semi-field validations are typically precision, robustness, linearity (concentration dependence), and range (reliable range of test values) [20, 23, 24, 34–36].

#### Define testing conditions

Test conditions encompass conditions critical for method performance. These can be identified from literature or in-house laboratory data [20]. Bioassay testing conditions include:

- Vector age: mosquitoes’ age during exposure affects knockdown and mortality outcomes [37–39].
- Vector status (sex, fed/unfed): male and female vectors respond differently to different insecticides [40] and the mosquitoes’ blood-feeding status impacts the post-exposure outcomes (e.g., mortality) [38] hence the mosquitoes’ sex and feeding status associated with the method claim should be specified.
- Preparation conditions for the vector, e.g., sugar starvation: nutritional status (blood or sugar-fed) of mosquitoes and the time mosquitoes are fed pre-exposure can alter the effects of insecticides [38, 41, 42].
- Vector holding conditions pre- and post-exposure: mosquitoes handling pre- and post-exposure may affect their metabolic and physiological status hence compromising the bioassay results. Also, mosquitoes should be given time to acclimatize to minimize bias [12].
- Time of day: time-of-day or whether experiments are conducted in the dark/light alters the mosquitoes’ metabolic detoxification and circadian rhythm hence influencing their responses to insecticide [43, 44]. Therefore, it is recommendable to conduct the tests at the same time of the day to minimize heterogeneity in the observed outcomes [12].
- Environmental conditions: other studies have shown that temperature (especially ambient) and relative humidity (RH) affect *Anopheles* mosquitoes’ larval development stages, resistance to insecticides, post-exposure outcomes such as mortality, longevity, blood-feeding digestion [12, 45, 46], fecundity, and infection rates [12, 47]. Also, the results can be influenced by larval rearing temperature, infection, density and feeding regime, and adult density in cages [12, 38, 41, 42, 48].
- Maximum/minimum number of vectors per replicate assay: number of vectors per replicate can influence the bioassay results observed [39].
- Sample handling conditions
  o Storage pre- and post-test
  o Time to reach ambient temperature prior to testing
  o Sample preparation (age, washing)

The way testing net samples are handled can influence bioassay results. For example, pyrethroids have temperature-dependent toxicity [49], and the effect of chlorfenapyr on mortality is positively associated with increasing temperature [50].

Standardized mosquito-rearing methods and testing conditions are crucial to obtain reliable, consistent, and reproducible results that are comparable within and across testing sites. To determine the optimum conditions for the method, experiments varying the testing conditions should be conducted, for example, changing the time of day that the bioassay is conducted to determine whether a mosquito’s circadian rhythm affects the results of the test [51]. A method is deemed robust if small variations in testing conditions do not heavily impact the performance of the method [25] for the purpose selected, e.g., evaluation of pyrethroid content on an ITN. Testing conditions may have a small or large impact on the assay results depending on the specific mode of action of the chemistry being bioassayed.

#### Select a *comparison* method (where applicable)

If the novel method has been designed to measure the same outcomes as an established standard method the new method should be compared to this. Standardized methods currently recommended in WHO guidelines are the WHO cylinder test and bottle bioassay to measure insecticide susceptibility, the WHO cone test and the tunnel test to characterize ITN fabrics, and Ifakara Ambient Chamber Test and experimental hut trials to measure entomological efficacy of ITNs [4, 33]. Select the method with the most similar test conditions and/or entomological endpoint(s) to the putative new method as the comparator method.

#### Define controls

Negative (baseline) and positive controls must be defined. Since it is not always known what non-insecticidal features of a product may impact the measured endpoint, the negative control should be as close as possible to the product under evaluation, e.g., a wall surface sprayed with water from the same source as will be used to mix the insecticide for an IRS method. The positive control should induce a known and significant impact on the endpoint under evaluation. Methods designed for characterization of dual AI products must include controls which contain each AI separately and in combination [52–54].

#### Conduct baseline and robustness experiments

Baseline experiments assess the performance of a method under assumed optimum testing conditions. Robustness experiments test the robustness of a method by identifying variables or testing conditions that might affect the method’s results [20, 55].

For baseline experiments:

(i) Consider the testing conditions that can potentially affect results and define standard measurement levels, for example a specified temperature range, to control for such effects
(ii) Conduct trial experiments using the simplest design possible, for example, tests using negative controls such as an untreated net for an ITN method
  For robustness testing:
(iii) Alter testing conditions or variables one at a time whilst keeping all other parameters unchanged. Although it is possible to vary multiple conditions simultaneously [20, 56], due to the high variability in bioassays, one variable at a time is recommended
(iv) Evaluate the degree of robustness: significance testing, a procedure used to quantify whether a result is likely due to chance or to some factor of interest, can be employed to determine the important factors for future consideration for assessing the method’s performance

##### Sample size

A sample size of at least twenty replicates per group should be used for baseline or robustness experiments [57].

When designing the experiments, apply the following definitions:

- Replicate: for example, a single set of five individual mosquitoes in a WHO cone test or mosquitoes exposed together in a Tunnel Test
- Sample: for example, a single piece of a net
- Testing system: for example, the mosquitoes being tested. Mosquitoes reared together under the same controlled conditions are referred to as the same testing system. This can be a single colony at a point in time or one colony maintained over time that is characterized and maintains fitness parameters within defined limits.

**Fig. 1:**
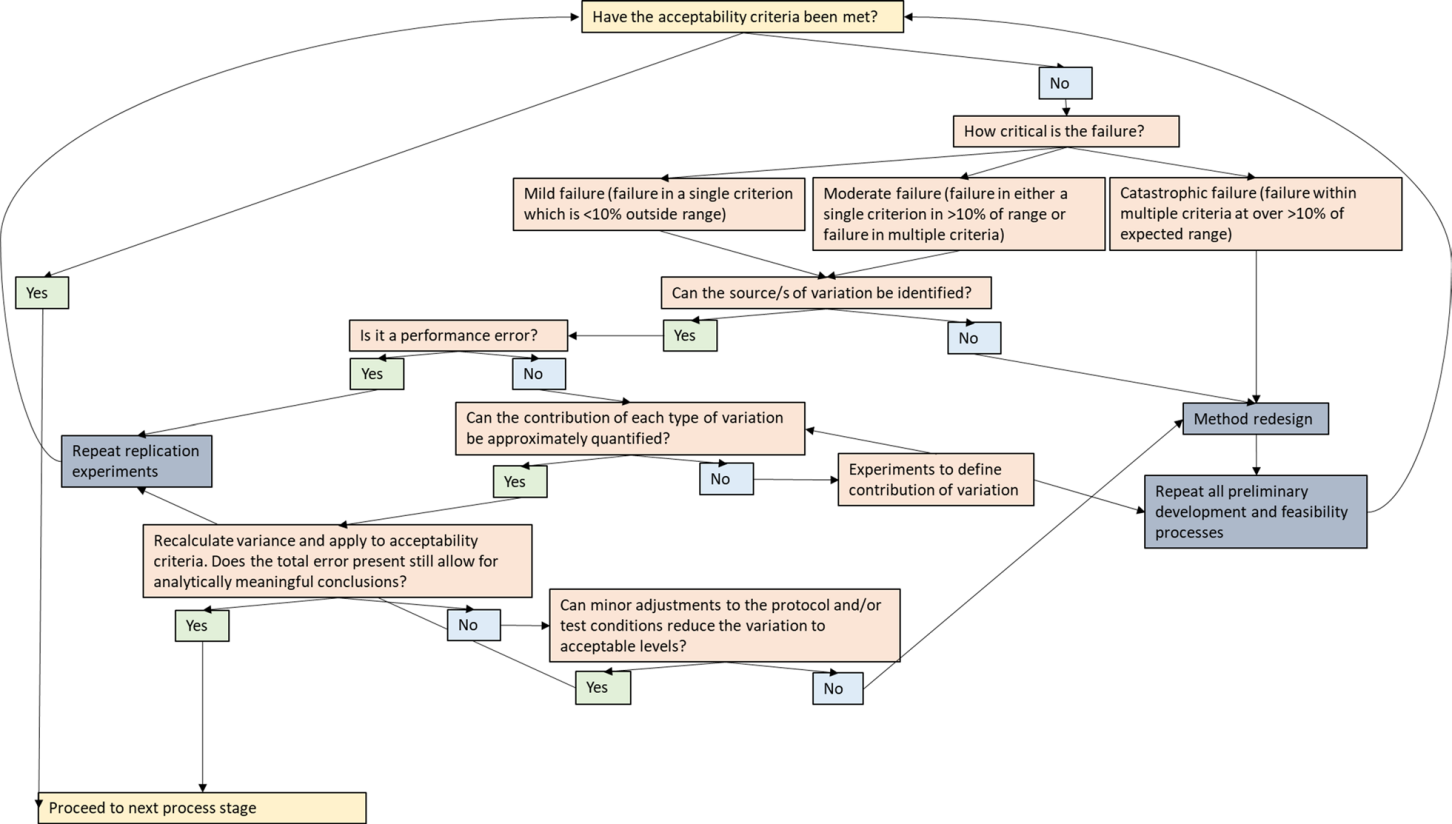
Decision tree for the evaluation of acceptability criteria following preliminary, feasibility, internal and external validation experiments

The data from the baseline or robustness experiments should be analysed and compared to the acceptability criteria. Where necessary the method can be modified, the outcome(s) and acceptability criteria refined and retested before proceeding to the feasibility stage. Figure 1 outlines a decision tree that can be used at each process evaluation stage to determine whether progression to the next stage is appropriate.

## Text box 1. Terminology

**Table:** 

• *Acceptability criteria* The allowable error within the method and are dependent on the effect size of each endpoint.	
• *Performance error* Errors that occur because of how the experiment is conducted, for example, not following or deviating from the standard operating procedures.	
• *Total error* The overall error in a test result that is attributed to imprecision and inaccuracy i.e. the net effect of random and systematic error in a method.	
• *Variability* Describes how far apart study/experiment data points lie from each other and from the center of a distribution.	
• *Variance* A measure of dispersion that takes into account the spread of all data points in a dataset/study. For more definitions of the method validation terminology refer to supplementary material (Additional file 1).	

### Feasibility experiments

Feasibility experiments are employed to understand the inherent variability of a method, to obtain values that can be used for estimating sample size for the internal validation experiments and to assess the utility and logistical ease of the proposed technique. Where two tests have equivalent performance characteristics, the one which is easier to use, cheaper, faster, more sensitive or more accurate might be preferred.

#### Estimating an appropriate sample size

Testing 20–30 replicates in a feasibility study is usually enough to obtain an estimate for variability/precision for use in formal sample size calculations [57, 58]. Ideally, 20–30 replicates in each study arm (WHO cones or cylinders, for example) would be tested on a single day to estimate within-day precision, followed by testing at least one replicate per day over a period of 20 days whilst holding all conditions constant to estimate between-day precision [22]. The sample size should be adjusted appropriately to suit the design of the method, performing at least four replicates per day. However, for bioassays that use long exposure times, such as the tunnel test, this study design is not possible and should be adjusted appropriately. Additionally, the use of insects as the test system in bioassays means that it is not possible to hold the test system constant, i.e., use the same mosquitoes each day. Rigorous colony rearing procedures should be followed to ensure colony stability to minimize insect variability, and data on fitness parameters should be collected for consideration as a potential source of variability [54]. To account for this variability, it is recommended that at least four replicates are tested for a minimum of five days wherever possible, and any analysis should include day of testing as a variable to account for the temporal bias inherent in bioassays using live insects (Additional file 1).

#### Describe testing pattern and testing period

The testing schema and testing period in the experimental design of feasibility experiments defines how the within and between-day error of the method will be measured, and are typically determined during replication sub-studies (refer to *Replication Studies*). The testing pattern should be balanced with respect to the number of replicates tested in a single day and the number of replicates tested each day over multiple days so that reliable estimates for the within and between-day precision are obtained.

#### Defining final endpoints for validation and drafting an SOP

During preliminary development and feasibility stages, multiple endpoints might be trialled. The data from feasibility studies is used to identify which of those endpoints are reliable and suitable for use in assessing the method’s performance during internal and external validation. All selected endpoints and their acceptability criteria should be included in a draft SOP.

#### Select strains for use in validation experiments

Both insecticide-susceptible and insecticide-resistant mosquito strains can be used in validation experiments. Where relevant, strains should be selected with reference to existing WHO testing guidelines [33], WHO implementation guidance, and published works. For example, Lees et al*.* [54] provides a strain characterization SOP which can be used for dual-AI ITNs and adapted as appropriate for other studies that require resistant mosquito strains.

### Internal validation

The purpose of the internal validation phase is to ensure that the method is reproducible within a laboratory, i.e., minimally validated, and to compile a data package that can be used by external laboratories/sites to externally validate the method.

#### Determining appropriate sample size and study design

Data from feasibility studies are used in a formal power calculation to determine the sample size for internal validation. This can be achieved by using standard formulas for sample size estimation or simulation studies for complex designs involving multiple varying factors and testing schema [59–61]. The predefined effect size for the primary endpoint of interest together with the SD/variability estimated from the feasibility experiments should be used to estimate the sample size. In a case where multiple endpoints are of primary interest, it is recommended that the endpoint with the smallest effect size and greatest variability in the feasibility experiments is used in the calculation [62].

#### Draft the method claim

This is a statement that clearly states the scope of the method, the outcomes, analytical parameters, and acceptability criteria associated with the method. Considerations to be taken into account when employing the method, for example, incorporating the variability of sample materials into sample size calculations, should be stated as part of the claim. For example, Video Cone Test (VCT) *PLUS,* an extension of the standard WHO cone test designed to characterize the effects of the co-formulations of pyrethroids and non-pyrethroid insecticides based on mosquitoes’ activity in the cone (imprecision/CV < 30%) and 24 h mortality within ± 3% the standard WHO Cone Assay. A detailed example of a method claim can be found on the Innovation to Impact (I2I) website [63].

#### Compile a data package

A data package must be produced by the laboratory that developed the method and provided to the external validating laboratories. The data package must include:

- SOP: step-by-step guidelines on how to perform the tests to ensure consistency in tests and reduce analytical errors. This includes the method background and purpose, equipment details and procurement information (if required), test protocol, all testing conditions, results analyses and interpretation, a list of known problems and their solutions, and any necessary quality control (QC) or calibration procedures (where applicable) for monitoring routine method performance.
  Ongoing QC procedures involve the comparison of control results against established reference ranges, and participation in external quality assurance (EQA) programmes. Laboratories and/or field sites acting as method validation centres should participate in (EQA) program at least twice per year.
- Method claim: states the scope of the method and is used to assess the performance of the method. This also ensures monitoring of the test’s validity and reliability over time in the same laboratory or across multiple sites. The required information include
  o Applicability
  o Measurement outcomes
  o Analytical parameters
  o Acceptability criteria and justification
- Study designs including sample sizes and testing schema: the standardized process allows direct and reliable comparison of the bioassay results from multiple sites. Also, this ensures that the experiments are properly powered for statistical analyses of the various outcomes and analytical parameters of interest.
- Define controls/criteria for selecting controls: enables proper assessment and understanding of the insecticide’s responses/effectiveness. Refer to the ‘Define controls section’ for the selection details.
- Criteria for strain selection: testing standardized mosquitoes across sites and/or characterization of resistant strains allows robust comparison and interpretation of results across tests performed within- and between- testing sites over time [54].

The method-developing laboratory should ensure that the product(s) and strain(s) used during the internal validation phase are characterized, and the results are provided together with the data package to assist with the interpretation of the validation results.

### External validation

As methods that use entomological endpoints to evaluate vector control tools are usually implemented in multiple laboratories and/or sites, to ensure reproducibility at least two external laboratories should validate the method [25, 64]. These laboratories extensively validate the method by ensuring that the method claim is reproducible at multiple sites/laboratories using a standardized SOP. This allows different levels of precision to be assessed, for example, within-day, within-laboratory, between-day and between-laboratory. The external validation sites should follow the experimental design proposed associated with the method claim that was defined following the internal validation stage. All the outcomes and analytical parameters associated with the method claim should be assessed.

A statement of the final claim and a full validation report is produced once external validation is complete. Ongoing quality assurance procedures or method verification in implementing sites certify results produced using the method.

### Validation sub-studies

**Fig. 2:**
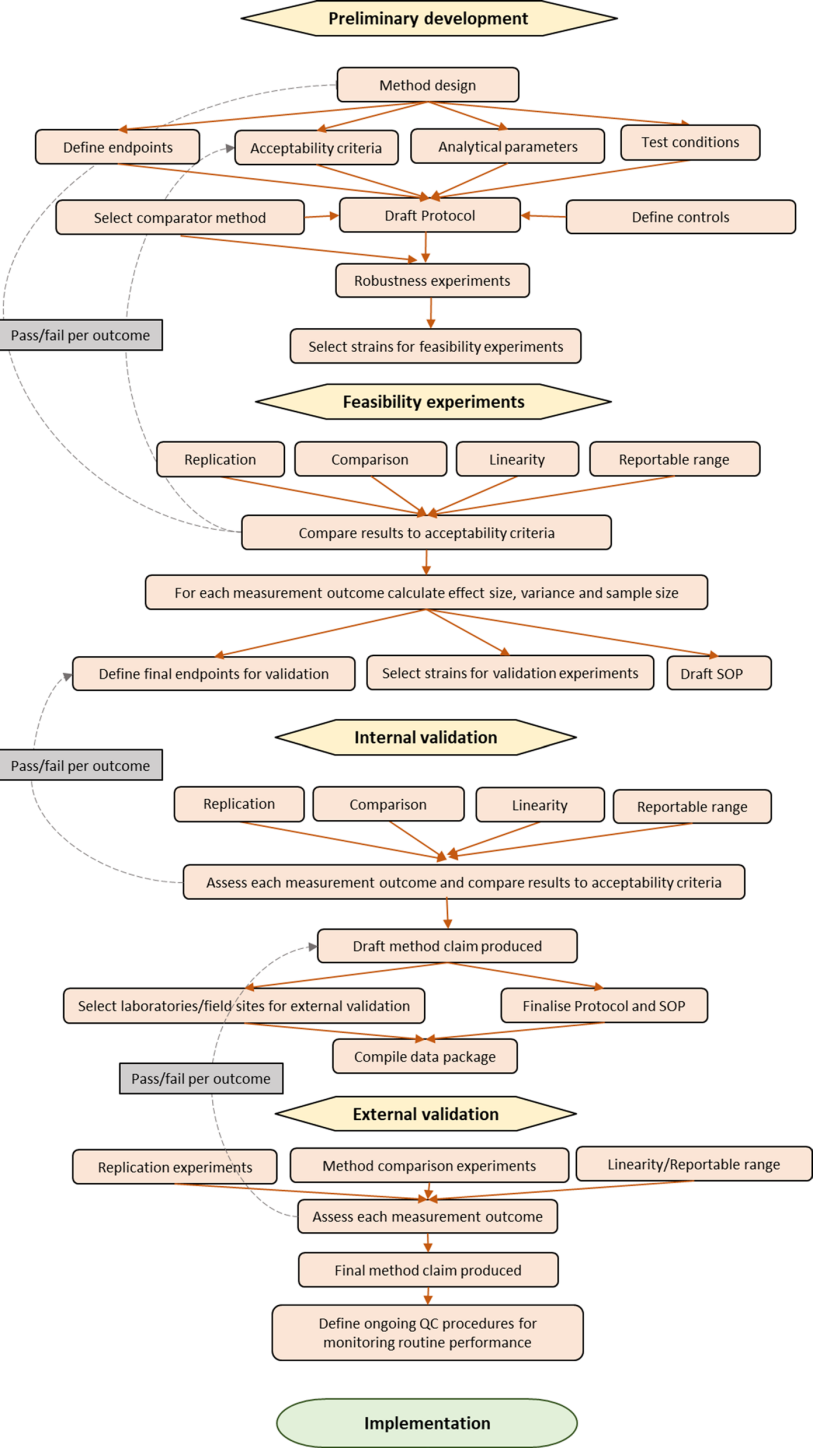
Method validation schematic

In each of the process stages of feasibility, internal validation and external validation, different relevant sub-studies are conducted depending on the intended purpose of the method and the design of the bioassay. Figure 2 shows each of the process stages and the possible sub-studies that might be employed.

When designing sub-studies, a single experiment can be designed for multiple purposes or to assess multiple analytical parameters. For example, an experiment designed to measure precision, i.e., a replication experiment, can include a comparator method, i.e., comparison experiments. Table 2 provides a summary of typical categories of the methods used to assess vector control tools and their associated studies and performance parameters.

**Table 2:** Examples of experimental types which could be applied as validation sub-studies for methods used to evaluate vector control products

Method purpose	Example/outcome	Sub-studies	Analytical parameters
Measure immediate lethality effects	• KD and 24 h mortality	• Define test conditions (P) • Replication • Comparison*	• Robustness (P) • Precision
Quantify downstream outcomes	• Delayed mortality (72–96 h) • Impacts on fecundity	• Define test conditions (P) • Replication • Comparison*	• Robustness (P) • Precision
Monitor success/failure of an outcome during exposure	• Blood-feeding success	• Define test conditions (P) • Replication • Comparison*	• Robustness (P) • Precision
Measure induced sterility	• Oviposition inhibition	• Define test conditions (P) • Replication • Comparison*	• Robustness (P) • Precision
Characterize behavioural responses during- and post-exposure	• Proportion of net contact time	• Define test conditions (P) • Replication • Comparison*	• Robustness (P) • Precision
Monitor durability	• Efficacy of ITNs after 3 years deployment • Duration of IRS efficacy	• Define test conditions (P) • Linearity and reportable range • Comparison*	• Robustness (P) • Linearity • Range • Precision
Evaluate combination products	• ITN with different concentration/s of AI/s on different parts of the net	• As per method purpose for each combination	• As per method purpose for each combination

*P* Preliminary development phase

^*^If applicable

#### Linearity or reportable range experiments

The reportable range of a method is the span of test values for which reliable results can be obtained; linearity is the ability of a method to obtain results, which are directly proportional to a given concentration [22, 64]. These studies can be implemented at all the stages of the validation process including baseline experiments. The purpose of these experiments is to determine a working range of the method’s results that is accurate and precise. For example, a reportable range for a method to measure the characteristics of an ITN might be the minimum to maximum level of 24 h mortality, which can be reliably measured by the method and the variability within the range.

For methods with phenotypic outcomes, establishing LD_50_ and/or LD_90_ for each active ingredient can be a substitute. Methods intended to be used for durability monitoring of products should be assessed using, e.g., ITNs that have undergone various numbers of washes, with accompanying chemical analysis of treatment concentration. This will approximate testing at different concentrations and ensure that method performance is validated against a range of different product conditions.

At least five replicates of known values at each concentration/number of washes (where appropriate) should be analysed by bioassay and chemical methods in triplicate to define the reportable range.

##### Data analysis

Linearity of the reportable range can be visually inspected using a scatter plot and line of best fit that fits the lowest points in the data series or fitting a regression line through the points in the linear range [22, 24, 65]. To control for potential confounding factors, the latter is recommended. For methods that are non-linear, a non-linear curve or a non-linear regression line can be fitted. For methods used to assess durability, precision should be evaluated throughout the range to determine the method’s reliability for estimating entomological outcomes over time/number of washes.

#### Replication experiments

Replication experiments are conducted during the feasibility, internal validation, and external validation process stages. During replication experiments, estimates are obtained for random error [22]. The goal is to determine the typical variability of the method during normal usage through measuring precision [25] and, therefore, the experimental design should encompass routine day-to-day variations.

Precision can be evaluated at different levels [25, 64]:

(i) Repeatability/intra-assay/within-run: precision observed among replicate bioassays performed under the same operating conditions within a day
(ii) Intermediate precision expresses within-laboratory variations: including different days, different operators, different mosquito-rearing cages.
(iii) Reproducibility: expresses the precision of agreement between laboratories

Repeatability and intermediate precision are evaluated during the feasibility and internal validation stages while all the levels of precision should be evaluated during the external validation. Repeatability variability is usually smaller compared to the other two levels of precision due to the many sources of variation that exist within and between laboratories contributing to the inter-laboratory variation than the within-laboratory or testing day [21, 25]. Therefore, careful attention should be paid when defining the acceptability criteria for the different levels of precision. Table 3 gives a summary of stages involved when conducting replication experiments.

**Table 3:** Replication experiments stages

Stage	Notes
1. Determine maximum number of replicates that can be performed in a single day	Four replicates are the minimum to calculate within-day imprecision. This number also accounts for potential outlying observations
2. Define total number of tests based on desired number of replicates and feasibility	Perform 20–30 replicates unless (Westgard, 2020) • Published data or • Preliminary development results indicate that 30 replicates are too few to evaluate measurement outcome Ensure sample size is sufficient to capture any downstream measurement outcomes, *e.g.,* fecundity Implement a balanced design where applicable. Here, the number of control replicates should equal the number of test replicates. If a balanced design is not practical, a larger proportion of the sample size should be allocated to treatment arms Refer to ‘Sample size’ section above for guidelines to determine sample sizes Replication experiments are performed for the entire defined reportable range (minimum 4 points with 20–30 replicates tested for each), where appropriate
3. Perform experiments over the smallest possible number of days	A minimum of four replicates should be tested per day for a minimum of 5 days in replication studies (holding all the testing conditions/factors constant) to allow estimation of the different levels of precision [21, 64] Where possible a balanced (equal number of replicates) design should be employed
4. Results from single days of testing are used to estimate within-run or day variation for each measurement outcome	If multiple test days were considered, pooled within-run or day variance estimates are be calculated
5. Results from multiple days of testing are used to estimate between-run or day variation for each measurement outcome	Calculate the between-days variance for the multiple testing days
6. Whole dataset provides estimate of variance for sample size calculations for total replication study	Used to estimate the total error for an entire experiment

##### Data analysis

Common measures for precision are SD or CV, also known as the relative standard deviation. However, these measures are not ideal if the data are non-normally distributed, contain a high proportion of outliers, and if the number of replicates per group are not equal [66]. In such cases, alternatives to the CV can be used, such as the Geometric Coefficient of Variation (GCV), Coefficient of Quartile Variation (CQV), Coefficient of Variation based on the Median Absolute Deviation (CV_MAD_), and Coefficient of Variation based on the Interquartile Range (CV_IQR_) for simple estimates [67–69] or the intra-class correlation coefficient (ICC) [70, 71]. More details about the formulas, pros and cons of each method, and examples of R packages (where possible) are contained in (Additional file 2: Table S1).

The data analysis performed should reflect the study design that was implemented, and a data analysis plan should be produced in advance alongside the study protocol. Usually, there are different sources of variation in replication studies, and it is important to estimate precision whilst accounting for the variability of all possible factors. These factors can be fixed and/or random variables, for example, estimating the within-day variability while accounting for the testing days, operators, and site variability. The most powerful approach for estimating precision for replication studies is using mixed-effects models, and the CV and/or ICC and their associated 95% confidence intervals (CI) [21, 70, 71]. The incorporation of 95% CIs is critical given the many unknown factors that can influence the results of a study but cannot be controlled for in the study design [62]. These analysis methods are applicable for various types of data including continuous, proportions, binary and counts. For example, this can be implemented by using the VCA (normal data only) and rptR R-packages among other software or packages [71, 72].

#### Comparison experiments

These experiments are conducted during the feasibility, internal validation and external validation phases and determine if there are any differences between an existing method and a new method. For example, the WHO cone test is the standard method to measure the impact of mosquito tarsal contact with an AI applied for vector control; a novel method developed to measure the impact of exposure using a different approach could be compared to the cone test to determine the comparability of the two methods. Usually, this is performed by testing the same sample by both methods [22]. However, such designs are not feasible for bioassays as the same insects/replicate samples cannot be used/measured twice using different methods/tests since preexposure will influence the outcome of a second exposure [5]. Therefore, comparison experiments for bioassays should be conducted in parallel using the same test system under the same conditions for both the pre-existing and novel methods to allow comparison.

Comparison studies for methods designed to evaluate products with new modes of action should be undertaken in parallel with a product of known performance using existing methods. Table 4 gives a summary for implementing comparison experiments.

**Table 4:** Comparison experiments

Stage	Notes
1. Select comparison method	
2. Determine the maximum number of replicates that can be performed in a single day	Four replicates are the minimum to calculate within-run imprecision
3. Perform 30 replicates each of new method and comparator method in parallel over the smallest possible number of days	Note that for methods with extremely high variation, 30 replicates may be insufficient

##### Data analysis

The data analysis will depend on the analytical parameter of interest, and it can be performed using the methods discussed above (as appropriate). To access the performance of the novel method, the Bland–Altman plot should be employed, to describe the agreement between the two methods based on the endpoint(s) of interest [73, 74]. The results obtained from the two methods should be compared within a group (i.e., holding all other conditions/parameters constant).

#### Measurement uncertainty

Validation results should be reported with an uncertainty measure (e.g., 95% CI), which indicates the margin of doubt that exists for the obtained results [25, 64]. For example, the CV as a measure for precision can be reported together with its corresponding 95% CI.

#### Outlying data points

Outlying data points/outliers are extreme values in an experimental dataset [72, 75]. Outliers can negatively impact results and/or the validity of fitted models by violating the normality assumption and therefore outliers should be identified and handled appropriately [21]. All extreme data points should be double checked to remove the possibility of recording error or operator error prior to outlier analysis. Outliers can be identified using visualization, e.g., boxplots, or formal statistical tests, such as the regression models or modified Grubb-test using the median and MD68-statistic, for example, this can be employed using the VCA R-package [72, 76]. The proportion of outliers should not exceed 1% in the total dataset [21, 72]. If outliers are identified, error estimates/analytical parameters such as precision should be calculated with and without the outliers to assess the impact of the outliers on the method’s performance results [21].

## Case studies

The case studies contained in Additional files 3, 4) contained worked examples that demonstrate the application of the concepts contained in the method validation framework at two different stages of the method validation process. Case Study 1 (Additional file 3) contains a description and worked example of the commercial validation of the Insecticide Quantification Kit, a test used to quantify residues applied to wall surfaces during IRS [77, 78]. This process contains concepts from the validation sub-studies of replication studies and limit of detection studies, and data analysis concepts of precision. The application of the decision tree (Fig. 1) is also described. Case study 2 (Additional file 4) contains the study design for the external validation of the VCT *PLUS*, an adaptation of the WHO cone test that is used to quantify mosquito behavioural responses to ITNs [79]. The process contains replication and comparison sub-studies and data analyses based on precision and to the 24 h mortality agreement between the VCT *PLUS* and WHO cone tests using the Bland–Altman plot.

## Discussion

The evaluation process for vector control products entering the market has evolved over time. Prior to the establishment of the vector control prequalification department in 2016 [80], responsibility for product assessment lay with the WHO Pesticide Evaluation Scheme (WHOPES), who conducted evaluation studies and developed testing guidelines [4, 5]. These processes relied heavily on the WHO cone bioassay, tunnel tests, and experimental hut trials (EHTs) to provide information on the effectiveness of insecticidal products [4, 5]. Recently, some groups have assessed the reliability of the standard tests, such as WHO cone and tunnel tests, and their findings suggest that the tests might not have been properly validated with unreliable results obtained when inherent stochastic variability and systematic bias are not accounted for [12, 39]. In addition, the lack of standard method validation guidelines for assessing novel methods resulted in ad hoc adoption of methods to characterize products even in the recognized absence of rigorous validation [81–85].

The new WHO ITN guidelines (currently in draft format), which are based on a “weight of evidence” approach require that quality data supporting an ITN listing should be produced using any appropriate test that the manufacturers deem suitable [33]. In this approach, appropriate method validation is paramount for building confidence in the method results and providing solid scientific evidence for its, and, by extension, the product’s, performance [20, 22, 23, 25, 64]. Ignoring the various sources of variability and/or not properly assessing bioassay and semi-field test precision could lead to misleading conclusions that inform future decisions [12, 61]. Therefore, a standardized approach to method validation that can be employed by manufacturers to ensure that the bioassays, tests and equipment used to evaluate vector control products are fit for purpose and reliable is necessary.

Unlike other fields where a method can be designed to be implemented in a single laboratory, the continuing processes of post-market testing, routine surveillance, and durability monitoring of vector control products necessitate that methods used to assess product characteristics are validated for implementation in a wide range of laboratories and testing sites. This complexity is increased by the range of product types used in vector control, e.g., ITNs, IRS treatments, repellents and larvicides, all of which might have different modes of action and applications, but which are united by the need to use entomological endpoints to characterize product characteristics [4, 5]. Additionally, the testing system may vary within and between laboratories over time, for example, colony differences of the same strain among testing sites due to differences in colony establishment, rearing conditions, selection pressures, genetic drift, contamination, and resistance maintenance hence limiting the possibility for robust comparison across sites and/or interpretation of results. Differences in laboratory capacity within and across sites, can potentially impact the study design (sample size and testing schema within and between testing days), and operator capacity [86] and/or vector attractiveness (for baited tests) [87] also influences test results. Following the validation framework described in this manuscript will ensure that methods are sufficiently sensitive to capture different modes of actions, can be used to assess the impact of new tools on various vector populations and allow comparability of results within and between testing facilities. Although the examples provided in this framework mainly focus on methods for evaluating product-based bioassays and semi-field studies, this approach can be extended to non-product-based assays with entomological endpoints, such as resistance monitoring bioassays.

It was not possible in this framework to provide standard cut-off points or acceptability criteria for various analytical parameters as are established in the chemistry or health sectors, due to the inherent variability in bioassays and the accepted variability in the specifications of the products that bioassays are used to characterize. Also, although some tests such as the WHO cone, tunnel tests and EHTs have been implemented for a long time, the focus of the experimental designs for these tests has been on satisfying the WHO pre-specified thresholds and not on assessing precision or robustness, hence historical data were judged to be unreliable for establishing standard acceptability criteria. However, methods on how best to establish suitable acceptability criteria for proposed methods have been provided.

The validation framework described in this report presents a more robust approach for assessing the performance of novel methods used to evaluate a product or tool than has been used previously, allowing a deeper understanding of the contribution of variability to each test method to be gained and delivering a more nuanced understanding of product characteristics and predicted effectiveness. Such understanding is vital given the continuing increase of insecticide-resistance in Afro-tropical malaria vectors and the demand for novel vector control chemistries or tools to combat this [88–90]. The adoption of this framework will ensure the rigorous evaluation of methods used to characterize vector control products, or vector populations, leading to timely and accurate data, and, ultimately, the appropriate deployment of the most efficacious products and tools to at-risk communities.

### Supplementary Information

**Additional file 1: **Glossary of standard method validation terms. This file contains the standard definitions for the terms used in method validation.

**Additional file 2: **Precision estimates. This file contains the different methods of estimating precision, their associated formulas, pros and cons, and examples of R packages for implementing the analysis.

**Additional file 3: **Case Study 1—Commercialization of the Insecticide Quantification Kit (IQK). This file contains a detailed example on how method validation can be implemented using this proposed framework.

**Additional file 4: **Case Study 2—Video Cone Test (VCT) *PLUS *Laboratory Assay. This file contains another example demonstrating how method validation can be implemented using this current proposed framework.

## Data Availability

Data sharing is not applicable to this article as no datasets were generated or analysed during the current study.

## Abbreviations

AI: Active ingredient
CI: Confidence interval
CV: Coefficient of variation
CQV: Coefficient of quartile variation
EHTs: Experimental hut trials
GCV: Geometric coefficient of variation
GLP: Good laboratory practice
I2I: Innovation to impact
ICC: Intraclass correlation coefficient
IRS: Indoor residual spraying
ITN: Insecticide-treated net
QC: Quality control
RH: Relative humidity
SD: Standard deviation
SOP: Standard operating procedure
VCT: Video cone test
WHO: World Health Organization
