# Supplementary material for: A bioassay method validation framework for laboratory and semi-field tests used to evaluate vector control tools
Source: Malar J. 2023 Sep 28;22:289. doi: 10.1186/s12936-023-04717-w (PMC10540336; doi:10.1186/s12936-023-04717-w)
Supplement: Supplementary file 1 — Additional file 1: Glossary of standard method validation terms. This file contains the standard definitions for the terms used in method validation. [file 12936_2023_4717_MOESM1_ESM.docx]

***Glossary of standard method validation terms***

**Accuracy:** The closeness of agreement between the mean value obtained from a large

series of test results and an accepted reference value.

**Acceptability criteria:** The allowable error within the method, which is dependent on the effect size of each endpoint.

**Allowable error:** The acceptable margin of error or measurement error requirements that must be met for experimental results to be fit for use.

**Analytical errors**: Errors that occur during the testing and result in increased variability in the data. These errors include equipment malfunction and operator error, for example, sample mix-ups, interference, undetected failure in quality control, and procedures not followed (i.e., deviations from standard operating procedures and assay instructions). For example, an operator wearing a strong scent when conducting assays on some testing days but not others which would interfere with mosquito attraction to a cue, or not testing controls for some testing days.

**Analytical parameter**: Parameter used to assess the performance of an analytical method. For example, robustness, precision, accuracy, etc.

**Analytical sensitivity:** Analytical sensitivity may be expressed as the limit of detection, i.e. the smallest amount of the target marker that can be precisely detected.

**Analytical specificity:** Analytical specificity means the ability of the method to determine solely the target marker.

**CV:** Coefficient of Variation

**GCV**: Geometric Coefficient of Variation

**CQV**: Coefficient of Quartile Variation

**CV_MAD_**: Coefficient of Variation based on the Median Absolute Deviation

**CV_IQR_**: Coefficient of Variation based on the Interquartile Range

**Effect size:** A quantitative measure of the magnitude of the observed effect, or effect of the intervention, on the endpoint.

**Evaluation:** A generic term to describe the measurement of the performance capabilities of a

system. This is a systematic and extensive process that compares different systems designed

to perform the same or similar functions. Where two tests have equivalent performance characteristics, the one

which is easier to use, cheaper, faster, more sensitive or more accurate might be

preferred.

**EQA:** External Quality Assurance

**False negative:** A specimen known to be positive for the target marker and misclassified by the device or testing system.

**False positive:** A specimen known to be negative for the target marker and misclassified by the device or testing system.

**Heterogeneity:** Differences in the testing system, study design, or variability in the intervention effects (i.e. inconsistent results) for the same outcomes being evaluated in the same or different studies.

**Intermediate precision:** expresses within-laboratory variations for results collected on different days, with different analysts, different equipment, etc.

**IQC:** Internal Quality Control.

**Linearity:** The ability of a method to obtain results which are directly proportional to a given concentration or level. This is employed when there is a linear relationship. Otherwise, Multi-point calibration can be employed for the methods that generate a non-linear response where curve fitting functions are considered to generate the calibration function.

**Measurement uncertainty:** A parameter associated with a measurement result that expresses the range of values that can reasonably be attributed to the quantity being measured.

Note: Measurement uncertainty is not a performance characteristic of a particular measurement procedure but a property of the results obtained using that measurement procedure.

**Method**: A method may be a new or modified bioassay, a technique using commercially available or in-house reagents, a prototype kit, a semi-field or field test, an in-house reagent or a set of reagents bought separately and used to prepare an in-house method.

**Method claim:** This is a statement that clearly states the scope of the method, the outcomes, analytical parameters, and acceptability criteria associated with the method.

**Operator error**: Errors introduced due to inaccuracies or mistakes by a person performing an experiment.

**Outlier:** Extreme values in an experimental/study dataset

**Performance error**s: Errors that occur because of how the experiment is conducted, for example, not following or deviating from the standard operating procedures.

**Precision:** A measure of the agreement for multiple measurements on the same sample, for example, replication experiments. Precision may be considered at three levels: 1) repeatability, 2) intermediate precision and 3) reproducibility. Usually, Repeatability ≤ Intermediate precision ≤ Reproducibility if the replicates are not too few or the time interval over which the experiments are conducted is not too short.

**Random error (imprecision):** an error that can be positive or negative, whose direction and exact magnitude cannot be predicted. Usually calculated by calculating the standard deviation (SD) from a set of replicate results. Calculating the percentage co-efficient of variation (%CV) from the SD gives a more understandable measure. The maximum size of random error is usually expressed as a 2SD or 3SD estimate. Random error often affects precision.

**Range of measurement:** The range of measured values for an outcome in which the acceptable or agreed error limits are not exceeded.

**Reliability:** Ability of a system or component to maintain performance within the developer’s or

manufacturer’s stated specifications over time.

**Repeatability:** Expresses the precision under the same operating conditions over a short interval of time. Repeatability is also termed intra-assay precision.

**Replicates:** Measurements of biologically distinct samples (e.g. the same type of organism treated or grown in the same conditions) that capture random biological variation, which can be a subject of study or a source of noise itself. For example, a single set of five individual mosquitoes in a WHO cone test or mosquitoes exposed together in a Tunnel Test.

**Reportable range:** The reportable range of a method is the span of test values for which reliable results can be obtained and for which it has been demonstrated that the analytical procedure has an appropriate level of precision, accuracy, and linearity.

**Reproducibility:** The ability to produce essentially the same result irrespective of variations in operator, test batch, laboratory or validated ancillary equipment, otherwise expressed as the precision between laboratories (in collaborative studies reproducibility usually refers to standardization of methodology).

**Robustness:** Capacity of an analytical procedure to remain unaffected by small but deliberate variations in method parameters, for example, temperature or time of day. This provides an indication of reliability during normal usage and repeatability when correctly used in multiple laboratories or settings. Robustness is synonymous with ruggedness.

**Run:** A set of experiments that are conducted in parallel with control experiments.

**Significance testing:** a formal statistical procedure for comparing observed data with a claim/hypothesis, that helps quantify whether a result is likely due to chance or to some factor of interest.

**SD:** Standard Deviation.

**Sensitivity:** The ability of an assay under evaluation to identify correctly true positive

samples, defined as samples that are positive in reference assays.

**Specificity:** The ability of an assay to identify correctly true negative samples, defined as samples that are negative in reference assays.

**Systematic error (inaccuracy):** an error that occurs only in one direction, e.g. a method consistently returns a value for an endpoint that is higher or lower than the ‘true’ value. The magnitude of systematic error is explained by calculating the bias, which is the average difference between the test method and a comparator method. In cases where a systematic error changes with the concentration of an analyte, it is referred to as a proportional error. Systematic error affects accuracy.

**True positive:** A specimen known to be positive for the target marker and correctly classified by the device or system.

**True negative:** A specimen known to be negative for the target marker and correctly classified by the device or system.

**Total error:** The overall error in a test result that is attributed to imprecision and inaccuracy i.e. the net effect of random and systematic error in a method on the results obtained.

**Validation**: A process that is used to demonstrate that a procedure is suitable for its intended purpose and that the results obtained are reliable.

**Variance:** A measure of dispersion that takes into account the spread of all data points in a dataset/study.

**Variability:** Describes how far apart study/experiment data points lie from each other and from the center of a distribution.

**Verification**: ISO 9000 (2005) defines method verification as *“confirmation, through provision of objective evidence, that specified requirements have been fulfilled”.* Verification occurs when a laboratory adopts a method that has already gone through basic validation, for example, a method that was already validated by a standards approving organisation or a method developed by a certain manufacturer. Before adopting the method, the end user’s laboratory is required to confirm their ability to produce acceptable results and the suitability of the analytical requirements of the method.
