# Supplementary material for: A bioassay method validation framework for laboratory and semi-field tests used to evaluate vector control tools
Source: Malar J. 2023 Sep 28;22:289. doi: 10.1186/s12936-023-04717-w (PMC10540336; doi:10.1186/s12936-023-04717-w)
Supplement: Supplementary file 2 — Additional file 2: Precision estimates. This file contains the different methods of estimating precision, their associated formulas, pros and cons, and examples of R packages for implementing the analysis. [file 12936_2023_4717_MOESM2_ESM.docx]

**Table S1. Precision estimates** [1–3]

| **No.** | **Estimate/Method** | **Pros** | **Cons** |
| --- | --- | --- | --- |
| 1 | Coefficient of Variation (CV):  $CV= \frac{SD}{Mean} \times100$   - SD = standard deviation - Commonly used to measure relative dispersion - Can be calculated by hand using the formula above or estimated from statistical models when estimating the different levels of precision is of interest using Analysis of Variance (ANOVA) or Restricted Maximum Likelihood (REML). This can be implemented using the VCA R package | - Easily applicable: Simple to calculate - Independent of the units of measurements - For completed designs (e.g., nested random factors): ANOVA or random-effects models can be employed | - Lack of robustness: prone to outliers - Not ideal for non-normally distributed data - Knowledge of mixed effects modelling required for complex designs - Not ideal when the number of replicates varies so much (simple calculations only) - Can be handled in random-effects models - Sensitive to small changes in the mean |
| 2 | Geometric Coefficient of Variation (GCV):  $GCV= \sqrt{exp( {SD}^{2}-1}) \times100$   - SD = standard deviation (log scale) - Can be estimated using the geocv function in PKNCA R Package | - An alternative to CV for lognormally distributed data - Suitable for percentages/ratio - Less sensitive to outliers than CV - Same as CV above | - Does not support zero or negative values - Prone to outliers compared to other estimates |
| 3 | Coefficient of Quartile Variation (CQV):  $CQV= \frac{(Q3-Q1)}{(Q3+Q1)} \times100$ | - Alternative robust estimator to CV in the presence of non-normally distributed data | - Difficult to employ for complicated designs (hierarchical/nested random effects) or correlated data |
| 4 | Coefficient of Variation based on the MAD (CV_MAD_):  ${CV}_{\mathrm{MAD}}=\frac{MAD}{Median} \times100$   - MAD = Median (\|Y_i_ – Median(Y_i_)\|) - MAD = Median Absolute Deviation | - Alternative robust estimator to CV in the presence of heavy-tail observations in the sample/skewed data - RCVM = 1:4826 x CV_MAD,_ comparable to CV under the normal distribution | - Same as CQV above |
| 5 | Coefficient of Variation based on the Interquartile Range (CV_IQR_):  ${CV}_{\mathrm{IQR}}= \frac{IQR}{Median} \times100$ | - Same as CV_MAD_ above - RCVQ = 0.75 x CV_IQR,_ comparable to CV the under normal distribution | - Same as CQV above |
| 6 | Intra-class Correlation Coefficient (ICC) /Repeatability (R)  $R\mathbf{=}\frac{VG}{VG+VR}$   - VG = group-level variance - VR = Residual variance - Based on the random effects models - rptR package in R: Normal, Binary, Proportion and Poisson distributions - ICC package in R | - Various distributions/types of data supported (proportions e.g proportion of mosquitoes dead at 24h in a WHO Cone/Tunnel tests; binary e.g whether each single mosquito fed or not at 1h post-exposure to a treatment/control using a WHO Cone test (data for each mosquito recorded separately); counts e.g. number of mosquitoes per night per sleeper in EHTs, etc) - Suitable for repeated/correlated measurements - Support complicated designs (estimate multiple grouping levels/random effects) - Supports unbalanced designs - Allows estimation of adjusted repeatability estimates (i.e., control for fixed effects) | - Knowledge of mixed effects modelling required: Cannot be estimated using descriptive summaries - Careful attention should be taken when designing the study (especially if the goal is estimate and evaluate different levels/sources of variability) |
| Note: Formulas for calculating the 95% confidence intervals (CI) for some of the simple estimates exist. However, bootstrap is recommended, especially with small sample sizes. | | | |

**References**

1. Arachchige CNPG, Prendergast LA, Staudte RG. Robust analogs to the coefficient of variation. J Appl Stat. 2022;49:268–90. <https://doi.org/10.1080/02664763.2020.1808599>.
2. Ospina R, Marmolejo-Ramos F. Performance of Some Estimators of Relative Variability. Front Appl Math Stat. Frontiers Media S.A.; 2019;5. <https://doi.org/10.3389/fams.2019.00043>.
3. Bower KM. Certain Approaches to Understanding Sources of Bioassay Variability - BioProcess InternationalBioProcess International. 2018 [cited 2023 Mar 17]. Available from: <https://bioprocessintl.com/upstream-processing/assays/certain-approaches-to-understanding-sources-of-bioassay-variability/>
