# Supplementary material for: A bioassay method validation framework for laboratory and semi-field tests used to evaluate vector control tools
Source: Malar J. 2023 Sep 28;22:289. doi: 10.1186/s12936-023-04717-w (PMC10540336; doi:10.1186/s12936-023-04717-w)
Supplement: Supplementary file 3 — Additional file 3: Case Study 1—Commercialization of the Insecticide Quantification Kit (IQK). This file contains a detailed example on how method validation can be implemented using this proposed framework. [file 12936_2023_4717_MOESM3_ESM.docx]

**Case Study 1 – Commercialisation of the Insecticide Quantification Kit (IQK)**
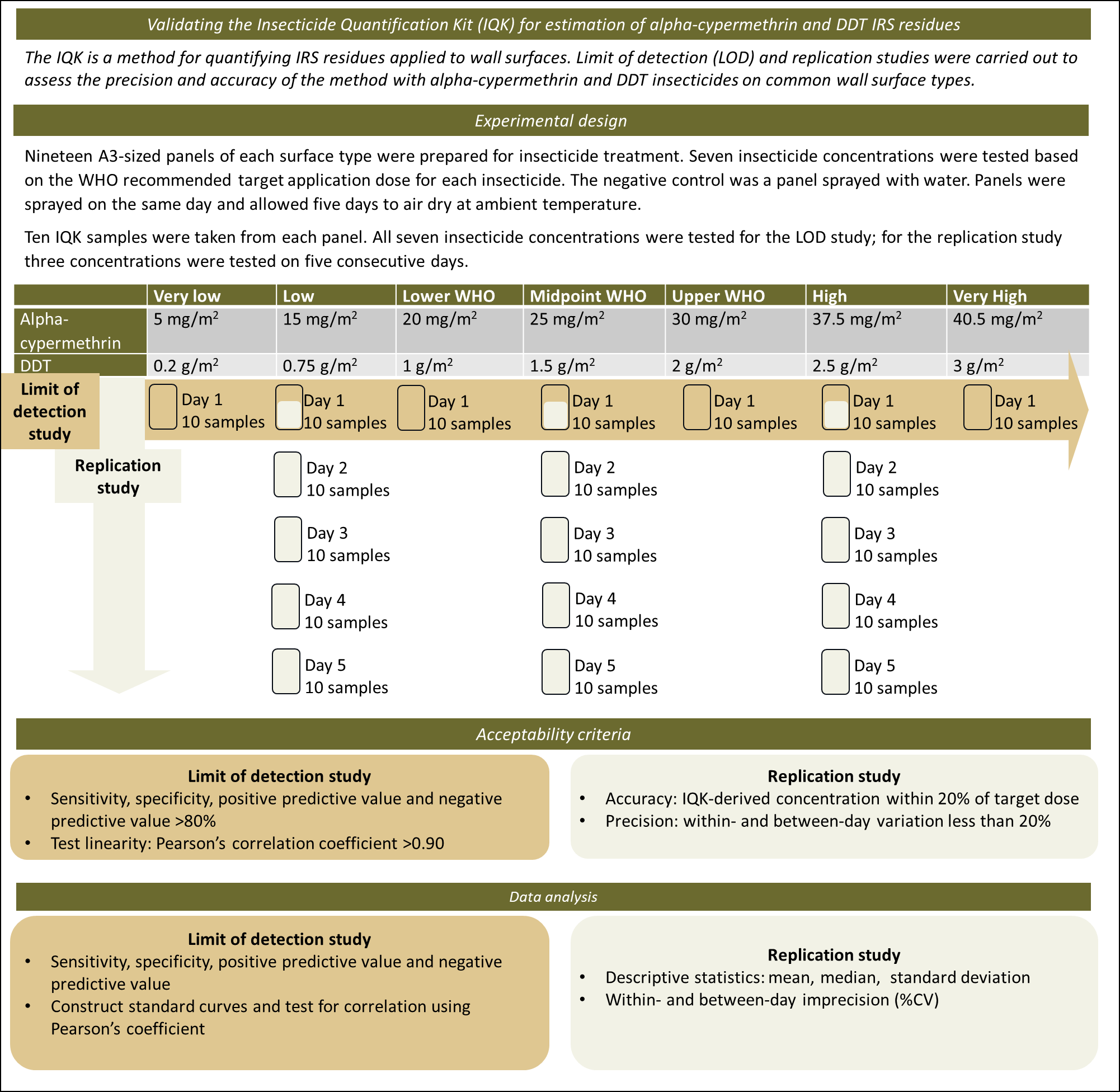


**IQK worked example**

***Commercial validation of the Insecticide Quantification Kit (IQK) for estimation of alpha-cypermethrin and DDT IRS residues***

***Background and purpose:***

The IQK is a method for quantifying the concentration of IRS residues on walls that uses a sticky spot or strip to lift insecticide from the wall surface. Following laboratory development and feasibility studies [1,2], two IQKs, the DDT and cyano-pyrethroid kits, were validated for commercial use using limit of detection (LOD) and replication studies were carried out to assess the precision and accuracy of the method with alpha-cypermethrin and DDT insecticides on common wall surface types.

***Wall surface selection:***

Wall surface types were selected based on common wall materials in countries using IRS. For the DDT IQK, walls were selected based on wall surfaces identified during site surveys in Bihar State, India [3]: bare brick, lime washed brick, mud, and thatch. For the cyano-pyrethroid IQK, surfaces were chosen based on common construction materials in sub-Saharan Africa: cement, mud and painted cement.

***Insecticide selection:***

Two insecticides were chosen. Insecticide selection was driven by programmatic use in the countries where the feasibility experiments were conducted [4], primarily Bihar State in India, where both DDT and alpha-cypermethrin were in use as part of the Visceral Leishmaniasis Elimination Programme.

***Insecticide concentrations:***

Twenty-four A3-sized panels of each surface type were prepared, nineteen for insecticide treatment and five negative control panels. To select the insecticide concentrations, the WHO recommended application dosage was used as a starting point for each insecticide. The lower and upper limits and the midpoint of each range were selected to ensure that the IQK could reliably quantify insecticide over the entire WHO recommended range. To test the limits of what the IQK could detect, four additional concentrations, two below the lower WHO limit and two above, were added to give a total of seven insecticide concentrations.

|  | **Very low** | **Low** | **Lower WHO** | **Midpoint WHO** | **Upper WHO** | **High** | **Very High** |
| --- | --- | --- | --- | --- | --- | --- | --- |
| Alpha-cypermethrin | 5 mg/m^2^ | 15 mg/m^2^ | 20 mg/m^2^ | 25 mg/m^2^ | 30 mg/m^2^ | 37.5 mg/m^2^ | 40.5 mg/m^2^ |
| DDT | 0.2 g/m^2^ | 0.75 g/m^2^ | 1 g/m^2^ | 1.5 g/m^2^ | 2 g/m^2^ | 2.5 g/m^2^ | 3 g/m^2^ |

***Panel preparation:***

DDT WP 75% (Hindustan Industries, India) and alpha-cypermethrin WP 5% were used to spray panels. Panels were sprayed using an 11L Hudson compression pump (Hudson, USA) pressurised at 40 -50 psi mounted in a rigid apparatus over a conveyor belt moving at 0.4 m/s. Panels were passed under the insecticide spray using the conveyor belt and allowed to dry at ambient temperature for five days before testing. Negative controls were sprayed with water. Positive controls non-sticky foil circles that were placed on panels and sprayed. All panels were treated simultaneously to avoid insecticide waste.

***Experimental design:***

The LOD and replication studies were conducted simultaneously over five testing days. All seven insecticide concentrations were tested for the LOD study; for the replication study three concentrations were tested on five consecutive days. Ten IQK samples were taken from each panel.


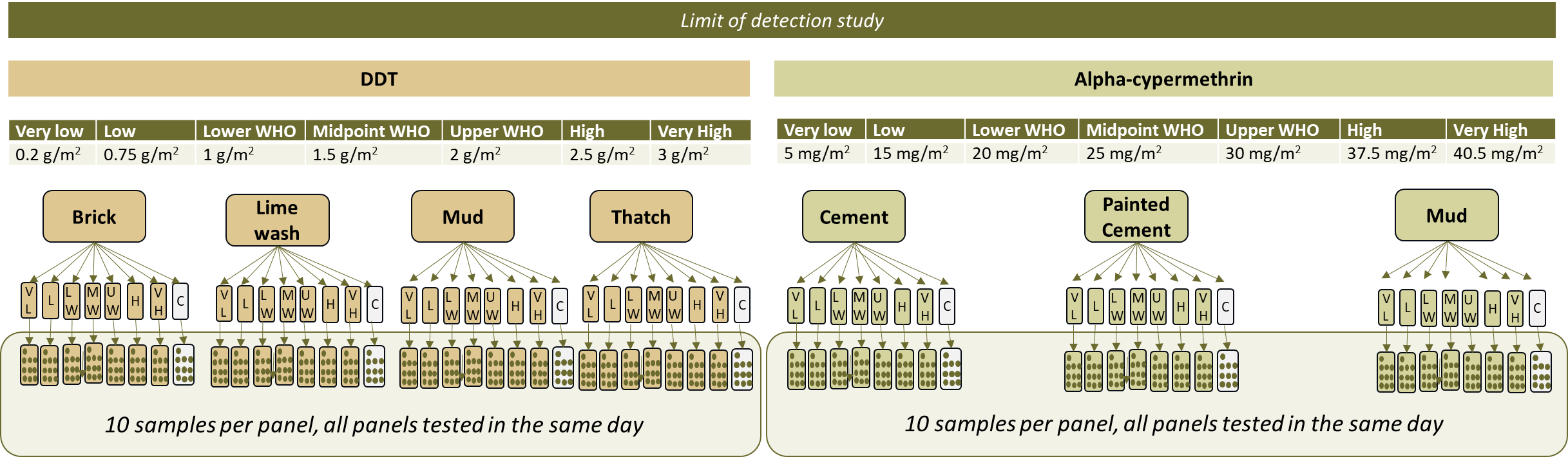


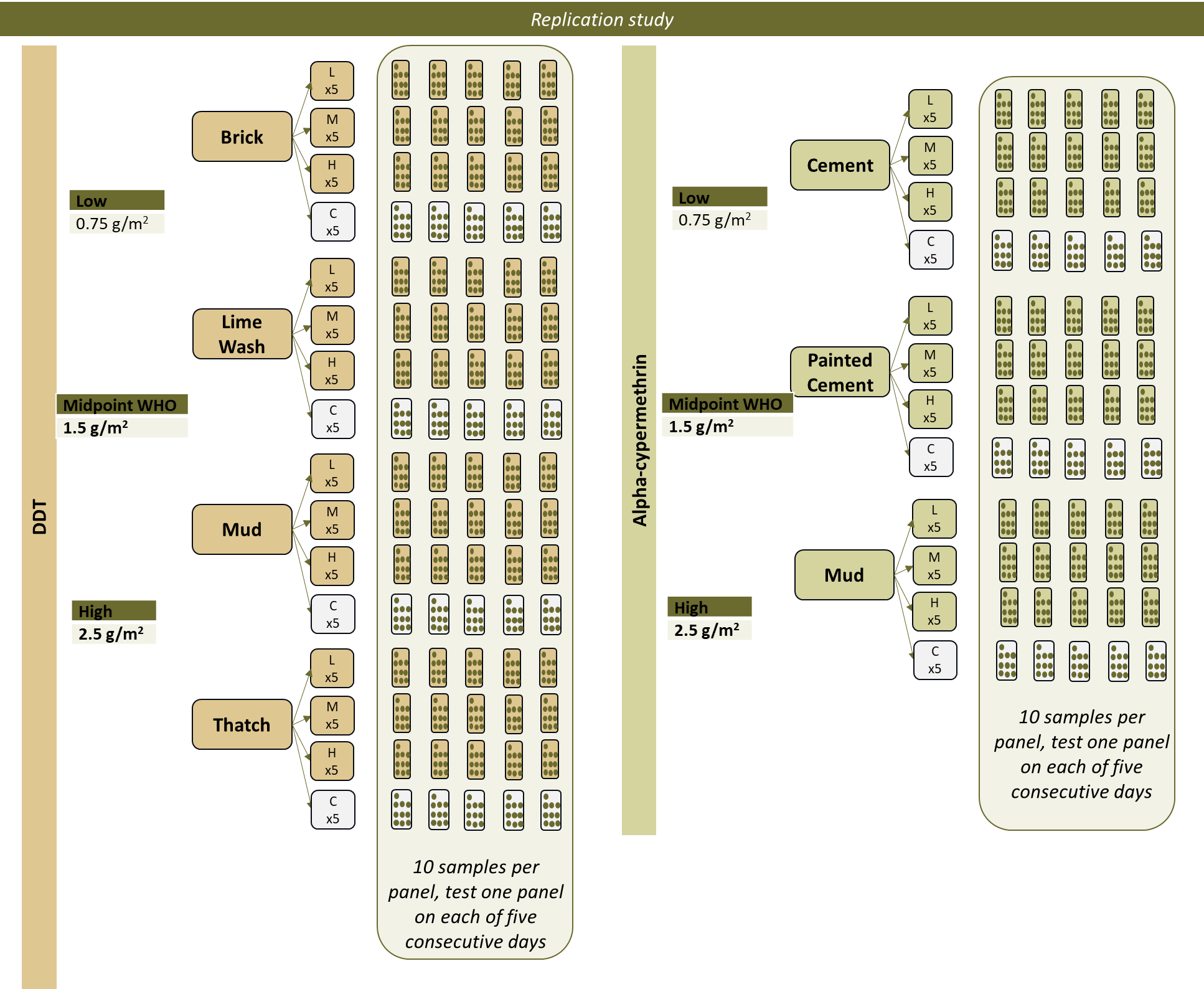


***Acceptability criteria:***

**Limit of detection study:** To assess the ability of the IQK to reliably distinguish the presence/absence of insecticide on sprayed surfaces, the specificity, sensitivity, positive predictive value and negative predictive values were calculated. The thresholds for these were set at 80%.

To evaluate the consistency of the IQK insecticide measurements over the entire tested range (0 – 3 g/m^2^ for DDT, 0 – 40.5 mg/m^2^ for alpha-cypermethrin). Standard curves were constructed and the correlation between the application concentration and the IQK-derived concentration was tested using Pearson’s correlation coefficient and non-linear regression. The threshold for acceptability was 0.90.

**Replication study:** To test the accuracy of the IQK-derived concentration, the mean and median IQK concentrations were compared to the target dose. The threshold set for accuracy was ±20% of the sprayed dose.

To test the precision of the IQK, the within-day and between-day variation was measured using the percentage coefficient of variation (%CV). The within-day variation was assessed for each of the five testing days. The precision threshold was %CV<20%.

***Data analysis methods used:***

Mean, median, standard deviation, %CV, sensitivity, specificity, positive predictive value, negative predictive value, non-linear regression, Pearson’s correlation coefficient.

***Results (1):***

In the limit of detection study, the sensitivity threshold (>80%) was breached for DDT/mud (69.4%), and the NPV threshold (>80%) was breached for all DDT surfaces and Alpha-cypermethrin/Mud.

|  | **DDT** | | | | **Alpha-cypermethrin** | | |
| --- | --- | --- | --- | --- | --- | --- | --- |
|  | **Brick** | **Lime Wash** | **Mud** | **Thatch** | **Cement** | **Painted Cement** | **Mud** |
| Sensitivity (%) | 85.7 | 93.6 | 69.4 | 92.5 | 98.2 | 98.8 | 90.7 |
| Specificity (%) | 100 | 95.0 | 90.0 | 100 | 100 | 100 | 95.2 |
| PPV (%) | 100 | 99.7 | 99.2 | 100 | 100 | 100 | 99.6 |
| NPV (%) | 27.0 | 44.2 | 13.4 | 48.8 | 83.3 | 87.0 | 41.7 |

In the replication study, the replication threshold (%CV<20%) was breached by the DDT IQK at at least one concentration per surface and at all alpha-cypermethrin surfaces and concentrations:

|  | **DDT between-day variation (%CV)** | | |  | **Alpha-cypermethrin between-day variation (%CV)** | | |
| --- | --- | --- | --- | --- | --- | --- | --- |
|  | **1.0 g/m^2^** | **1.5g/m^2^** | **2 g/m^2^** |  | **15 mg/m^2^** | **25 mg/m^2^** | **37.5 mg/m^2^** |
| Brick | 53.4 | 16.0 | 14.9 | Cement | 35.3 | 25.5 | 23.6 |
| Lime Wash | 33.4 | 25.2 | 27.7 | Painted Cement | 45.6 | 62.2 | 55.7 |
| Mud | 340.8 | 26.7 | 23.6 | Mud | 77.0 | 49.3 | 37.4 |
| Thatch | 23.5 | 18.3 | 16.8 |  |  |  |  |

Using the decision tree, both studies had multiple failures greater than 10% outside the expected range, classifying both as suffering catastrophic failure. Method redesign was undertaken to increase the surface area of each sample and the LOD and replication studies were repeated.


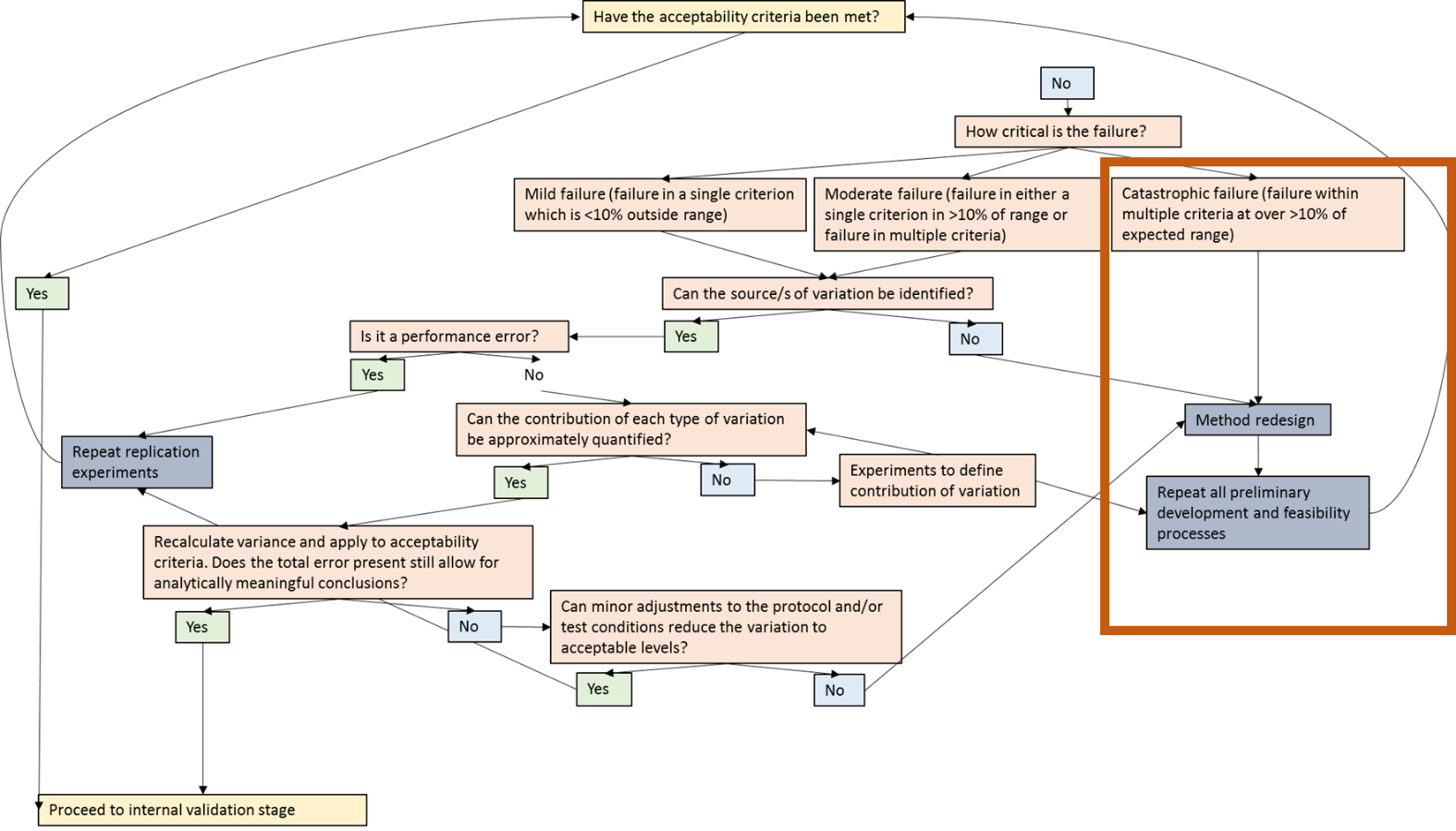


***Results (2):***

**Limit of detection study:** Sensitivity, specificity, PPV and NPV were calculated for all surfaces individually and combined. In the limit of detection study, the sensitivity, specificity and PPV thresholds were passed for all surfaces and insecticide concentrations. The NPV threshold for DDT was improved compared to the initial LOD study but did not meet the threshold and the NPV for Alpha-cypermethrin/painted cement did not pass the threshold, although the combined results for all surfaces did.

|  | **DDT** | | | | | **Alpha-cypermethrin** | | | |
| --- | --- | --- | --- | --- | --- | --- | --- | --- | --- |
|  | **Brick** | **Lime Wash** | **Mud** | **Thatch** | **Combined results** | **Cement** | **Painted Cement** | **Mud** | **Combined results** |
| Sensitivity (%) | 96.3 | 94.7 | 96.3 | 90.5 | 94.5 | 100 | 96.9 | 100 | 98.9 |
| Specificity (%) | 100 | 100 | 90.0 | 100 | 100 | 100 | 100 | 100 | 100 |
| PPV (%) | 100 | 100 | 99.2 | 100 | 100 | 100 | 100 | 100 | 100 |
| NPV (%) | 58.8 | 50.0 | 58.8 | 35.7 | 48.8 | 100 | 62.5 | 100 | 83.3 |

The decision tree was used for investigatory work. The failure in the DDT/NPV thresholds was due to false negative results in the 0.2g/m^2^ concentration. No false negative results were observed in other concentrations. Based on this, the lower limit of detection of the DDT kit was set at 0.75g/m^2^, the next lowest concentration tested. Testing with a new range of concentration between 0.2g/m2 and 0.75g/m2 to determine the precise lower limit of detection for the kit was ruled out at the time but remains a possibility for the future should a more precise limit be required.

*Test linearity*

Test linearity was calculated for all surfaces combined and individually. The Person’s correlation coefficient for DDT was 0.90 for combined surfaces and 0.85 for brick, 0.97 for lime wash, 0.95 for mud, and 0.91 for thatch, all within the acceptability threshold. For alpha-cypermethrin, the correlation coefficient was 0.92 for combined surfaces and 0.99 for cement, and 0.92 for painted cement and mud, all within the acceptability criteria.


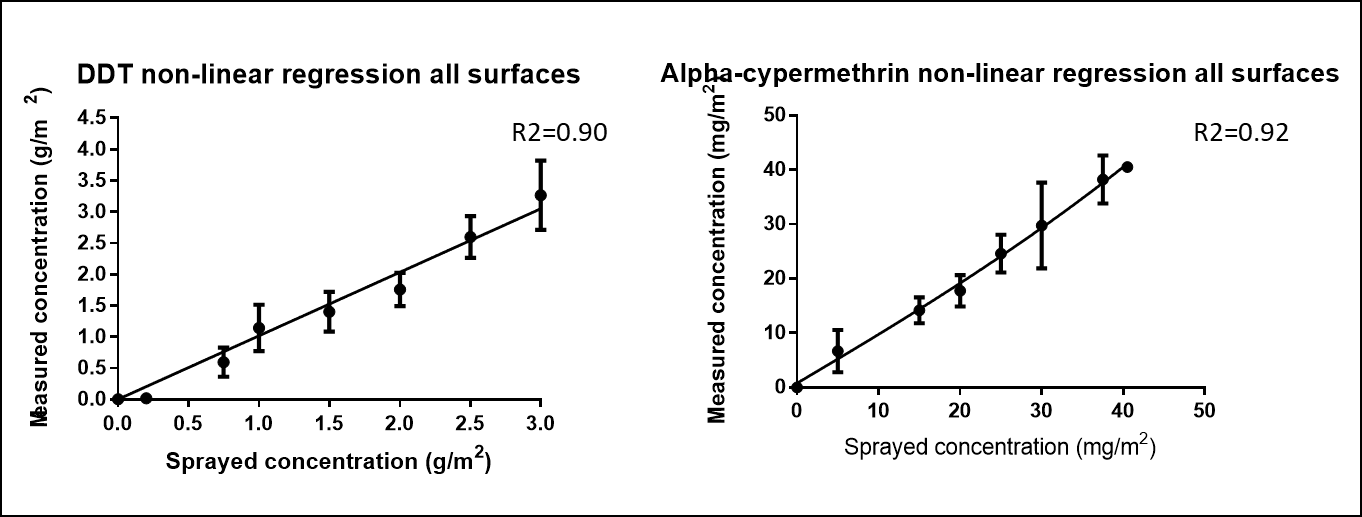


**Replication study:**

*Precision:*

In the replication study, the within- and between day variation was calculated for all surfaces individually and combined. For DDT, the combined between-day replication threshold was breached for the 0.75g/m^2^ and 1.0 g/m^2^ concentrations, particularly for thatched surfaces. Difficulties in sampling from springy thatched surfaces using the sticky sampling method were identified.

For alpha-cypermethrin, the between-day replication threshold was breached for the lowest concentration of cement and painted cement. The deviation from the threshold was less than 10%. The within-day variation was <20% for all cement and mud test days/concentrations. For painted cement tests, two test days showed >20% variation at the 15mg/m^2^ concentration (Day 1 %CV = 25.22, Day 3 %CV = 21.30) and the 25 mg/m^2^ concentration (Day 4 %CV = 22.36, Day 5 %CV = 26.19); all test days for the highest concentration passed the precision threshold. Two of these days showed greater than 10% deviation from the threshold.

|  | **DDT between-day variation (%CV)** | | |  | **Alpha-cypermethrin between-day variation (%CV)** | | |
| --- | --- | --- | --- | --- | --- | --- | --- |
|  | **0.75 g/m^2^** | **1.0g/m^2^** | **2.5 g/m^2^** |  | **15 mg/m^2^** | **25 mg/m^2^** | **37.5 mg/m^2^** |
| Brick | 35.29 | 16.78 | 9.31 | Cement | 21.75 | 15.68 | 8.38 |
| Lime Wash | 14.03 | 7.37 | 1.49 | Painted Cement | 21.37 | 17.49 | 10.88 |
| Mud | 20.11 | 8.63 | 4.23 | Mud | 7.13 | 6.01 | 1.49 |
| Thatch | 59.30 | 22.31 | 13.99 | Combined | 21.87 | 15.07 | 8.18 |
| Combined | 48.38 | 21.20 | 12.67 |  |  |  |  |

The decision tree was used to investigate the results. For the alpha-cypermethrin cement surface, each individual day passed the <20% variation threshold, but the between-day variation was >20%, suggesting either fluctuations in environmental conditions or in the panels that were affecting test results (note that as all panels were sprayed at the same time, differences in the treatment applied to each panel could be eliminated as a cause). Each result was plotted chronologically, and a degradation in the amount of cement recovered from panels in Days 4 and 5 was observed. This accounted for the increased between-day variation observed. When the results for Test days 4 and 5 were excluded from the between-day analysis, the result was within the <20% threshold.


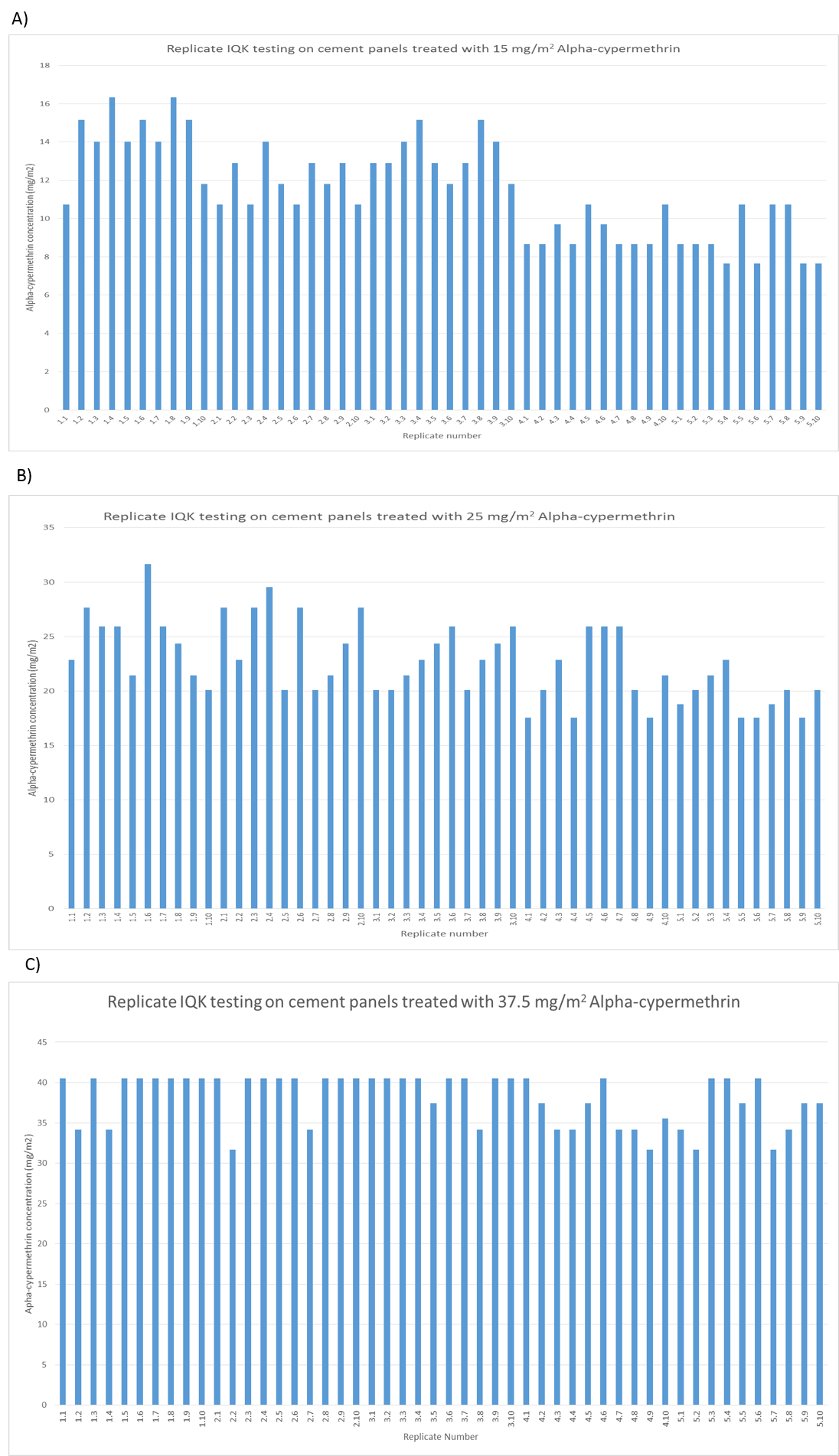


For the painted cement surface, notes from experimental records were consulted to identify the source of variation. It was noted that the sticky sampling circles frequently lifted a layer of paint from the surface of the panel, which did not separate during the extraction process. This would account for the greater variation observed.

*Accuracy*

The accuracy of the IQK was calculated for each surface individually and for all surfaces combined. The mean and median of the DDT combined results were within 20% of the target dose and passed the accuracy threshold. By surface type, each surface/concentration combination passed the accuracy threshold with the exception of brick/1.5 g/m^2^ (±20% range = 1.2-1.8 g/m^2^, mean brick = 1.15, median cement = 1.10).

The mean and median of the alpha-cypermethrin combined results were within 20% of the target dose and passed the accuracy threshold. By surface type, each surface/concentration combination passed the accuracy threshold with the exception of the lowest concentration of alpha-cypermethrin on cement and painted cement (±20% range = 12-18 mg/m^2^, mean cement = 11.54, median cement = 11.27, mean painted cement = 10.79, median painted cement = 11.61). The deviations from the threshold were within 10%.

The degradation observed on cement surfaces and the variability caused by the sticky sampling tool identified during the precision study were judged to be the cause of the threshold breaches. When the accuracy was recalculated using only the days with no observed degradation, the accuracy thresholds were passed.

***Validation decision:***

Following the LOD and replication studies, and taking into account the variability of some of the observed results, the IQK kits were validated for commercial production with the following caveats:

- To accurately quantify the application delivered during an IRS campaign, samples should be taken from walls within seven days post-spray;
- The sticky sampling tool may life painted from painted surfaces; if so, variability in the results is to be expected;
- The nature of thatched surfaces creates difficulties in sampling that affects the consistency of results;
- The lower limit of detection for the DDT IQK is 0.75g/m^2^, the lower limit of detection for the alpha-cypermethrin kit is 5mg/m^2^;
- In all other circumstances the IQK performs within the acceptability criteria of ±20% of the delivered insecticide dose.

***References***

1. Ismail HM, Kumar V, Singh RP, Williams C, Shivam P, Ghosh A, et al. Development of a Simple Dipstick Assay for Operational Monitoring of DDT. PLoS Negl Trop Dis. Public Library of Science; 2016;10:e0004324. <https://doi.org/10.1371/journal.pntd.0004324>.
2. Russell TL, Morgan JC, Ismail H, Kaur H, Eggelte T, Oladepo F, et al. Evaluating the feasibility of using insecticide quantification kits (IQK) for estimating cyanopyrethroid levels for indoor residual spraying in Vanuatu. Malar J. BioMed Central Ltd.; 2014;13:1–8. <https://doi.org/10.1186/1475-2875-13-178>.
3. Coleman M, Foster GM, Deb R, Singh RP, Ismail HM, Shivam P, et al. DDT-based indoor residual spraying suboptimal for visceral leishmaniasis elimination in India. Proc Natl Acad Sci USA . National Academy of Sciences; 2015;112:8573–8. <https://doi.org/10.1073/pnas.1507782112>.
4. World Health Organization. Kala-Azar elimination programme: report of a WHO consultation of partners, World Health Organization. Geneva, Switzerland; 2015 Feb.
