# Supplementary material for: A bioassay method validation framework for laboratory and semi-field tests used to evaluate vector control tools
Source: Malar J. 2023 Sep 28;22:289. doi: 10.1186/s12936-023-04717-w (PMC10540336; doi:10.1186/s12936-023-04717-w)
Supplement: Supplementary file 4 — Additional file 4: Case Study 2—Video Cone Test (VCT) PLUS Laboratory Assay. This file contains another example demonstrating how method validation can be implemented using this current proposed framework. [file 12936_2023_4717_MOESM4_ESM.docx]

**Case Study 2 – Video Cone Test (VCT) *PLUS* Laboratory Assay
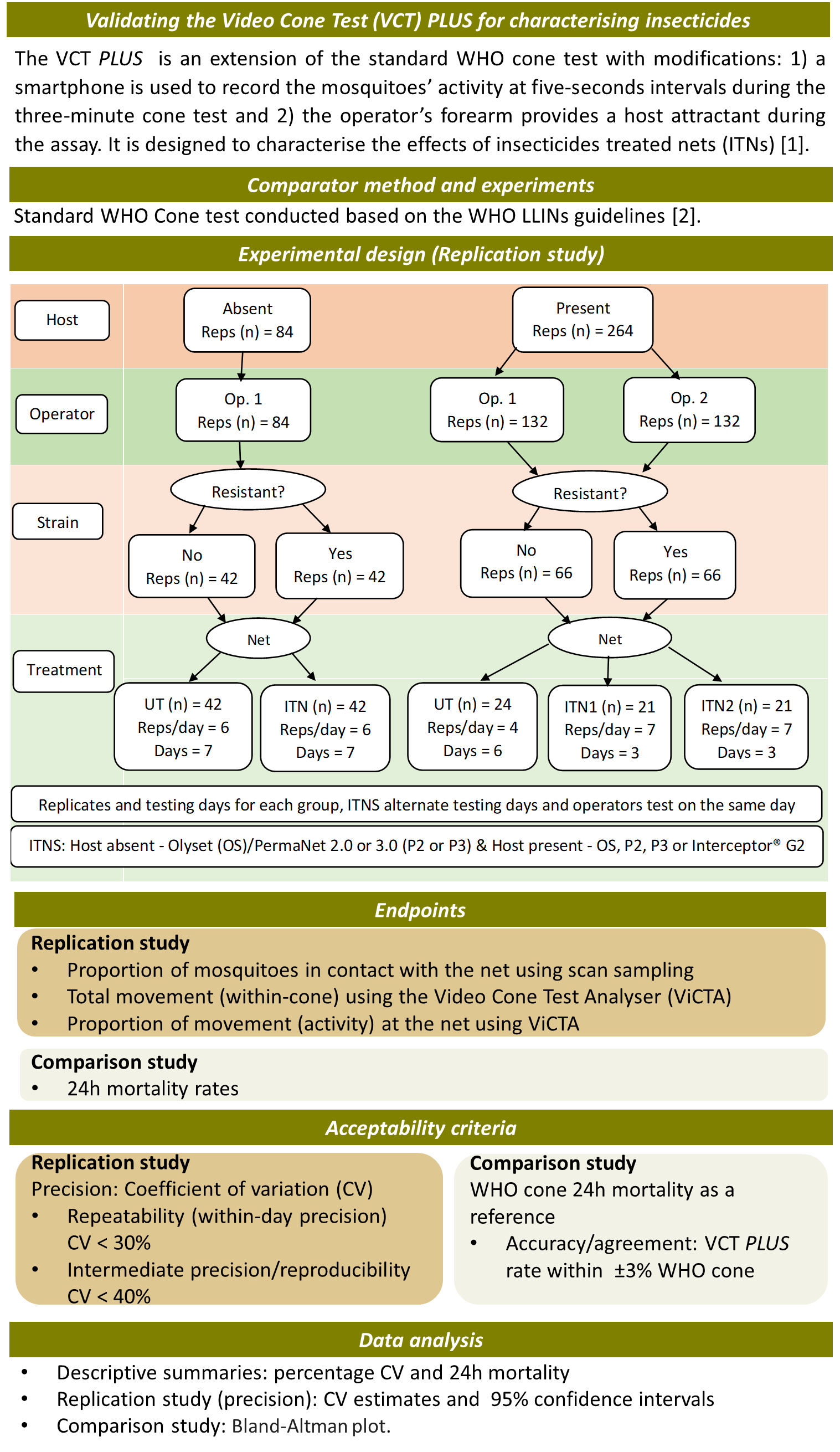
**

**References**

1. Hughes A, Matope A, Emery M, Steen K, Murray G, Ranson H, et al. A closer look at the WHO cone bioassay: video analysis of the hidden effects of a human host on mosquito behaviour and insecticide contact. Malar J. BioMed Central Ltd; 2022;21:1–11. <https://doi.org/10.1186/s12936-022-04232-4>.
2. WHO, WHO Pesticide Evaluation Scheme. Guidelines for laboratory and field testing of long-lasting insecticidal nets (No. WHO/HTM/NTD/WHOPES/2013.1). 2013.
